# Intravenous immunoglobulin therapy in erythromelalgia management: a case report

**DOI:** 10.31744/einstein_journal/2025RC1236

**Published:** 2025-02-26

**Authors:** Renato Ádler Pomilio de Sousa, Luydson Richardson Silva Vasconcelos, Marcus Villander Barros de Oliveira Sá

**Affiliations:** 1 Internal Medicine Department Real Hospital Português de Beneficência Recife PE Brazil Internal Medicine Department, Real Hospital Português de Beneficência em Pernambuco, Recife, PE, Brazil.; 2 Instituto Aggeu Magalhães Fundação Oswaldo Cruz Recife PE Brazil Instituto Aggeu Magalhães, Fundação Oswaldo Cruz, Recife, PE, Brazil.

**Keywords:** Erythromelalgia, Immunoglobulins, intravenous, Small fiber neuropathy, Autoimmune diseases, NAV1.7 Voltage-gated sodium channel

## Abstract

A 49-year-old woman presented with a 16-year history of burning pain, warmth, redness, and edema in both toes, feet, legs and calves. Despite extensive medical testing, including genetic analysis, no specific cause was identified. Initial treatments failed to improve symptoms, leading to impaired quality of life and mental health. Eventually, a six-month course of intravenous immunoglobulin therapy provided complete relief, allowing the patient to resume normal activities. Erythromelalgia is a rare neurovascular condition characterized by pain, warmth, and erythema in the extremities. It can manifest as primary, inherited or sporadic, or secondary to underlying conditions, such as hematological neoplasms. Although genetic studies suggest a pivotal role of a gain-of-function mutation in the Nav1.7 voltage-gated sodium channel in familial cases, the pathogenesis underlying sporadic adult-onset cases remains uncertain. The frequent coexistence of autoimmune connective tissue diseases and the expanding evidence supporting immunotherapies in idiopathic small-fiber neuropathies underscores the possible involvement of adaptive immunity in such conditions. Given the potential complications in untreated patients, risks associated with long-term opioid therapy, and the absence of disease-modifying strategies, intravenous immunoglobulins may offer a more effective approach to pain control than conventional pain relievers, representing a promising direction for understanding the pathogenesis of erythromelalgia.

## INTRODUCTION

Erythromelalgia is a rare neurovascular disorder that manifests as a triad of pain, erythema, and elevated extremity temperature.^1-3^ Various classification schemes have been proposed, but most authors have separated erythromelalgia into two main categories: primary and secondary. The secondary form is a complication of a predisposing condition, notably myeloproliferative disease, and tends to resolve with treatment of the underlying disorder.^1^ In contrast, the primary form engenders more debate.

Categorizing a patient with primary erythromelalgia (PE) alone indicates no identifiable causative factors. Some prefer to subdivide this group into two clusters: inherited early- childhood/adolescence-onset and sporadic late- or adult-onset diseases.^4-6^ There is a strong association between inherited cases and gain-of-function mutations in the *SCN9A* gene, which encodes the Nav1.7 voltage-gated sodium channel (VGSC).^7,8^ This channel depolarizes small-fiber neurons, particularly unmyelinated nociceptive C-fibers. Conversely, patients who develop symptoms in adulthood usually lack a clear etiopathogenesis.

## CASE REPORT

**Figure 1:**
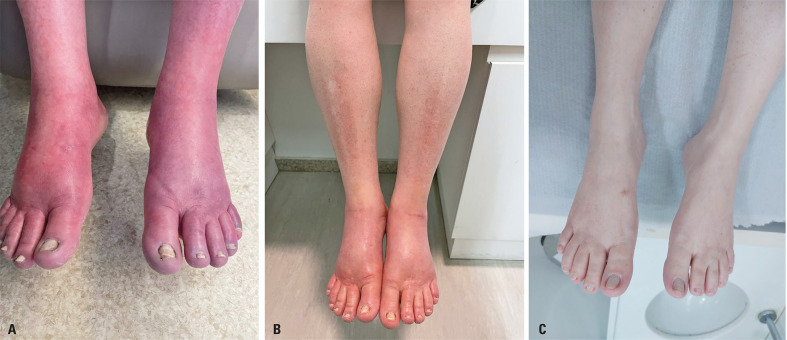
Result of IV immunoglobulin therapy. Visual aspect of the patient’s legs before (A), right after (B), and 18 months after (C), a six-month course of intravenous immunoglobulin treatment directed against erythromelalgia

A 49-year-old woman presented to our office with a 16-year history of burning pain, warmth, redness, and edema in both toes, feet, legs and calves. Her medical history was significant only for the presence of systemic arterial hypertension and glucose intolerance. No clear trigger could be identified, as some pain crises occurred even during sleep, and cold water immersion of the lower limbs provided only partial pain relief. Symptoms progressively worsened over the years, severely limiting her functional abilities (Figure 1A) and leading to mental illness, which prompted psychiatric treatment for depression and pathological anxiety.

Incisional skin biopsy of the right ankle revealed nonspecific vascular changes. Despite the absence of other connective tissue signs, nail fold capillaroscopy revealed moderate capillary dilation, occasional microbleeds, and capillary entanglements, with no evidence of devascularization. Electromyography indicated sensory axonal polyneuropathy and suggested small fiber prevalence. Comprehensive testing for antinuclear antibodies, anti-neutrophil cytoplasmic antibodies, complement, cryoglobulins, antiphospholipid antibodies, rheumatoid factor, anti-CCP, anti-SSA, anti-SSB, anti-Jo1, anti-RNP, anti-centromere, anti-Scl70, parvovirus, Epstein-Barr virus, cytomegalovirus, and serology for human immunodeficiency virus, hepatitis B virus, and hepatitis C virus, and Lyme disease, yielded negative results. The erythrocyte sedimentation rate, C-reactive protein, serum and urine protein electrophoresis, aldolase, CPK, cyanocobalamin, folic acid, thyroid-stimulating hormone (TSH), and glycohemoglobin (HbA_1C_) levels were within normal limits. Several ultrasonographic examinations of the lower limbs with arterial and venous color Doppler revealed no abnormalities.

To diagnose PE, genetic testing was performed, including JAK2 mutation and a genetic panel for sensory neuropathies encompassing 11 genes: *ATL1*, *DNMT1*, *FAM134B*, *IKBKAP*, *KIF1A*, *NGF*, *NTRK1*, *SCN9A*, *SPTLC1*, *SPTLC2*, and *WNK1*. No mutations were identified in any of these analyses.

The patient received an extensive array of treatments to alleviate her symptoms. Initially, the patient was prescribed aspirin, pregabalin, gabapentin, lidocaine patches, carbamazepine, and pentoxifylline at therapeutic doses. As her condition progressed, corticosteroids, methotrexate, and hydroxychloroquine were administered. However, no improvement was achieved. Given the sustained resistance to symptoms, intravenous immunoglobulin (IVIg) therapy was initiated. The patient received 400 mg/kg daily for five days, and this cycle was repeated every 30 days for six months. Her symptoms entirely resolved at the end of treatment, allowing her to resume normal activities, including work (Figure 1B). Eighteen months later, there was no sign of recurrence (Figure 1C).

The study was approved by the research ethics committee of the *Real Hospital Português de Beneficência em Pernambuco*, CAAE: 79729224.6.0000.9030; # 6.859.621.

## DISCUSSION

Recent literature has attempted to analyze erythromelalgia within the context of broader syndromes and has suggested a role for immune dysfunction, particularly in acquired cases previously considered idiopathic.^8-10^ The frequent coexistence of autoimmune connective tissue diseases such as systemic lupus erythematosus and Sjögren’s syndrome underscores the hypothesis that innate and adaptive immunity generate neuropathic pain.^9^

Just as hyperfunctioning VGSCs promote neurogenic inflammation and neuropathic pain through C-fiber hyperexcitability,^4,8^ hypofunctioning voltage-gated potassium channels (VGKC) may elicit the same effect. VGKCs belong to the ion channel family, responsible for the repolarization and inactivation of sensory nerves. Ellwardt et al.^9^ asserted that while genetic influences predispose individuals to pain primarily during early life, there may also be acquired dysfunction of VGKCs, leading to pain in individuals without an apparent predisposition. This statement is supported by evidence such as the discovery of inactivating autoantibodies against VGKCs and their coupled proteins (*e.g*., anti-VGKC, anti-CASPR2, and anti-LGI1).

Regardless of its etiopathogenesis, the diagnosis of PE relies on the clinical history and physical examination; no definitive treatment has yet been established.^1^ The most commonly used drug options include aspirin, nonsteroidal anti-inflammatory drugs (NSAIDs), β-blockers, antihistamines, and vasodilators. Still, none appear to improve symptoms or quality of life (QoL) significantly.^4^ Its association with markedly reduced QoL scores and survival rates compared with age- and sex-matched control subjects^4^ emphasizes the need to explore new therapeutic possibilities.

Given the potential complications in untreated patients, the risks associated with long-term opioid therapy, the absence of disease-modifying strategies, and the growing body of evidence supporting the use of IVIg (Table 1),^6,9,10-12^ it is reasonable to consider that immunotherapy may be a more effective approach for pain control than conventional pain relievers, highlighting a promising direction for understanding the pathogenesis of erythromelalgia.

**Table 1:** Evidence from the experimental use of IVIg in erythromelalgia

Case reports	Clinical information	Interventions and results
Jackson et al. 2008^(6)^	A 64-year-old female with SAH presented with burning pain and purplish erythema in her hands and feet, along with thrombocytopenia (consistent with ITP), cyanocobalamin deficiency, and positive anti-parietal cell antibodies, with no evidence of anemia. During follow-up, she was also diagnosed with CAD	The first intervention with 4 mg/day of methylprednisolone led to partial improvement, and she was then administered IVIg (400 mg/kg) for three consecutive days, yielding marked improvement in pain and discoloration two months later. Eventually, symptoms resumed three months after the first IVIg infusion, and she was prescribed a second session in higher doses (500 mg/kg for 3 days). Seven months after the second dose, she remained in remission
Ellwardt et al., 2020^(9)^	A 33-year-old female presented with a one-year history of erythema, edema, and burning/stinging pain in her hands, chest, and legs. These symptoms were associated with vertigo, headaches, memory problems, visual disturbances, and intermittent sinus tachycardia. Comprehensive testing revealed pleocytosis, elevated CSF total protein, and positive serum anti-CASPR2 autoantibodies. This case is one of three involving small fiber neuropathy due to anti-CASPR2 syndrome, with the patient diagnosed explicitly with erythromelalgia	The first intervention with corticosteroids and IVIg elicited no improvement. Monthly plasmapheresis then led to a return of symptoms to the baseline. Eventually, the patient did not return for follow-up appointments
Bourkas et al., 2023^(10)^	An 18-year-old female with GERD, atopic march, eosinophilic esophagitis, hyperhidrosis, milk protein allergy, *Spina bifida occulta*, and childhood asthma presented with acute chest pain due to esophageal microperforation, which was treated conservatively. Eventually, she developed GI spasms with flushing and burning pain in her lower limbs, face, and chest	Over 13 months, the patient underwent immunotherapy, starting with 1 g/kg of IVIg every 3 weeks. This led to improved extremity temperature and GI symptoms but caused side effects like migraines and nausea, prompting a change to 20 g weekly. After six months, side effects prompted a switch to 24 g of SCIg weekly, divided into 12 g twice weekly. After eight months of immunotherapy, the patient could resume her university studies
Rey et al., 2003^(11)^	A 66-year-old female was treated for ITP with IVIg and prednisolone, with a good response. Seven months later, she had a relapse of ITP. Concomitantly, she noticedthe occurrence of erythromelalgia with paroxysmal burning pain and redness of the distal extremities	Erythromelalgia was treated with a second course of IVIg and associated prednisolone, leading to symptom regression. However, after tapering the corticosteroid dose, the pain resumed. A splenectomy was then performed, and there was no recurrence of ITP or erythromelalgia 18 months later
Moody et al., 2012^(12)^	An 18-year-old female with a strong family history of autoimmune disease presented with a 6-year history of numbness and red-purple discoloration of her limbs, face, and neck, triggered by minor traumas. This was associated with seronegative polyarthritis, recurrent hordeola, and episodes of dysphasia	Administration of two doses of intravenous immunoglobulin (1 g/kg per dose, 48 hours apart) reduced the frequency and severity of her pain episodes from attacks lasting two weeks every two months to two- to three-day attacks occurring every six months

Available case reports in the medical literature on the experimental use of intravenous immunoglobulins for erythromelalgia are scarce, heterogeneous, and lack standardization; however, they present promising results.

CAD: Coronary Artery Disease, CAPSR2: Contactin-Associated Protein 2; CSF: Cerebrospinal Fluid, GERD: Gastroesophageal Reflux Disease, GI: Gastrointestinal, ITP: Immune Thrombocytopenia, IVIg: Intravenous Immunoglobulin, SAH: Systemic Arterial Hypertension, SCIg: Subcutaneous Immunoglobulin.
